# The complete mitochondrial genome of Ternate False Fusus *Brunneifusus ternatanus* Gmelin, 1791 (Neogastropoda: Buccinoidea: Melongenidae) obtained using next-generation sequencing

**DOI:** 10.1080/23802359.2021.1942270

**Published:** 2021-06-21

**Authors:** Hongtao Liu, Mingqiu Yang

**Affiliations:** a Key Laboratory of Utilization and Conservation for Tropical Marine Bioresources (Hainan Tropical Ocean University), Ministry of Education, Sanya, China; b Hainan Provincial Key Laboratory of Tropical Maricultural Technologies, Hainan Academy of Ocean and Fisheries Sciences, Haikou, China

**Keywords:** *Brunneifusus ternatanus*, mitochondrial genome, phylogenetic analysis

## Abstract

In this paper, the complete mitochondrial genome of Ternate False Fusus *Brunneifusus ternatanus* Gmelin, 1791 was determined and characterized for the first time from the South China Sea. Our results showed that the length of the whole mitogenome of *B. ternatanus* was 16,254 bp and the mitogenome consisted of 22 tRNA genes, 13 protein-coding genes (PCGs), and 2 rRNA genes. Furthermore, the nucleotide composition of this mitogenome is significantly biased with G + C contents of 31.85% (the base content was 30.38% A, 16.17% G, 37.77% T, and 15.68% C). All PCGs shared ATG as the initiation codon, and stop codon of TAA or TAG, with the exception of *COX2* which ended with a single T. The phylogenetic tree showed that *B. ternatanus* was first clustered with *Hemifusus tuba*, and from a single distinct cluster. The new complete mitochondrial genome provides new insight into the phylogenetic of Melongenidae and its evolution.

*Brunneifusus ternatanus* Gmelin, 1791, also known as Ternate False Fusus, is a species of sea snails, marine gastropod mollusks in the family Melongenidae. The scientific name, *B. ternatanus*, has only recently been accepted (previously named *Hemifusus ternatanus*) as a result of emerging research (Dekkers 2018). The characteristic features of *B. ternatanus* are similar to *Hemifusus tuba*, but it is longer and slenderer, usually smooth and without angular whorls, with a longer siphonal canal which is about 2/3 portion of the shell. *Brunneifusus ternatanus* is widely distributed in Western Pacific, especially in Japan (Phillips and Depledge 1986), Singapore (Chan 2009), Malaysia (Vermeij and Raven 2009), and China. It is found in the subtidal deep-water zone at a depth of 10–70 m. Because they are sold at a high price in markets, it has caused their overfishing. The taxonomy and phylogeny of the Melongenidae even Buccinoidea have been debated due to the lack of reliable morphological taxonomic characters (Kantor 2003). Here, we report the new complete mitochondrial genome of Melongenidae, which will provide a better insight into phylogenetic assessment and taxonomic classification.

The specimens of *B. ternatanus* were collected from Huanqiu fishing port of Wenchang, China (N19°33′51.12″, E110°49′27.98″), and deposited in the Hainan Marine Science and Technology Museum (Hongtao Liu, xmulht@gmail.com) under the voucher number S20201203BT in Qionghai research base of Hainan Academy of Ocean and Fisheries Sciences. The *de novo* DNA libraries of *B. ternatanus* had an average length of 350 bp were constructed using the NexteraXT DNA Library Preparation Kit, and sequencing was performed on the Illumina Novaseq platform (Total Genomics Solution Limited, SZHT) generating reads of 150 bp in average length. The complete mitochondrial genome of *B. ternatanus* was assembled with 4.35 G clean reads using the de novo assembler SPAdes 3.11.0 (Bankevich et al. 2012) and was annotated using the MITOS (http://mitos.bioinf.uni-leipzig.de/index.py). Based on 13 protein-coding genes (PCGs) encoded by 45 species mitogenomes available in the GenBank, the phylogenetic analysis was carried out using IQ-TREE v1.6.12 (Nguyen et al. 2015) with the maximum-likelihood (ML) method used to investigate the evolution position of *B. ternatanus.* The bootstrap replicate was set as 1000, the best-fit model was chosen mtMet + F+R5 according to Bayesian information criterion (BIC).

The whole mitogenome of *B. ternatanus* is 16,254 bp in size (GenBank Accession No. MW548267). The overall base content was 30.38% A, 16.17% G, 37.77% T, and 15.68% C respectively. The 68.15% of (A + T) showed a high AT bias. The sequences of the *B. ternatanus* mitogenome consist of 22 tRNA genes, two rRNA genes, and 13 protein-coding genes (PCGs). Eight tRNA genes were located on the light strand, the others were encoded by the heavy strand.

The 22 tRNA genes in the mitogenome of *B. ternatanus* vary in length from 62 bp to 71 bp. There were two type copies of tRNA-Leu and tRNA-Ser, respectively, in the mitogenome. The 12S rRNA was 889 bp long and located between tRNA-Val and tRNA-Glu. The 16S rRNA was 1386 bp long and located between tRNA-Val and tRNA-Leu. All 13 PCGs shared a common initiation codon ATG. Simultaneously, 12 PCGs were terminated with the common stop codon TAA or TAG, except *COX2* ended with a single T. There are 2 overlapping regions of 1–7 bp in length, and the longest overlapping region is located between *ND4* and *ND4L*. The mitochondrial genome has 25 intergenic sequences varying from 1 to 935 bp in length. The largest intergenic sequence is located between tRNA-Phe and *COX3* (Table S1).

The phylogenetic tree (Figure 1) showed that *B. ternatanus* formed a single distinct clade by clustering with *Hemifusus tuba*, and further clarified the phylogenetic relationships of the species of Melongenidae in the order Neogastropoda. Our phylogenetic result of *B. ternatanus* is consistent with the previous study using nuclear gene (18S rRNA and segment histone H3) and mitochondrial gene (*COI*, 12S rRNA, and 16S rRNA) (Zou et al. 2011). Taken together, the whole mitochondrial genome of *B. ternatanus*, characterized here, serves to clarify the phylogenetic relationships and comparative mitogenome studies of Melongenidae species in the Neogastropoda.

**Figure 1.**
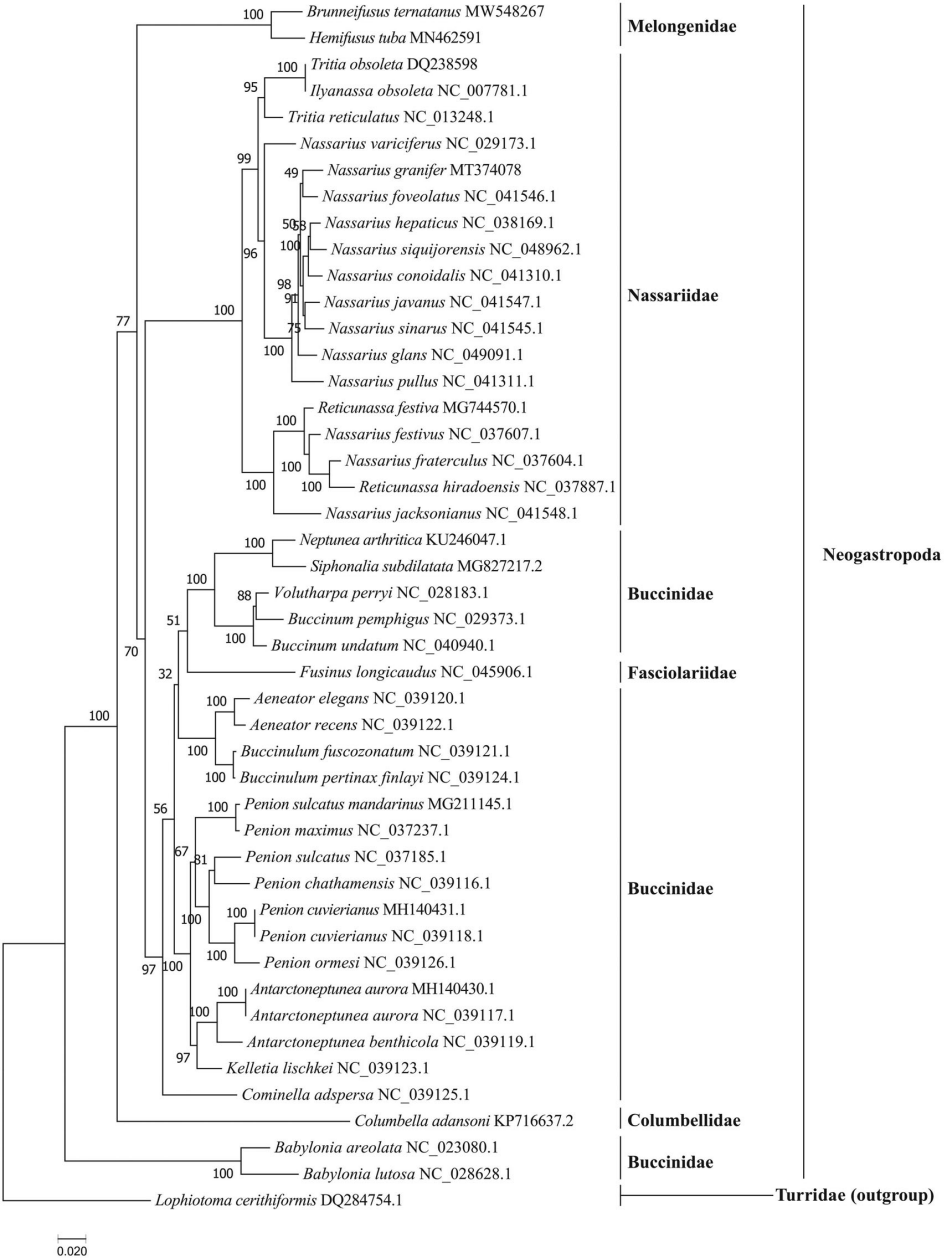
The maximum-likelihood tree of *B. ternatanus* and 45 other species based on 13 PCGs.

The GenBank accession number for each species is indicated after the scientific name. The ML tree was carried out using IQ-TREE v1.6.12 with 1000 bootstrap replicates, the best-fit model is mtMet + F+R5. The bootstrap values were labeled at each branch node. *Lophioyoma cerithiformis* was used as an outgroup.

## Data Availability

The genome sequence data that support the findings of this study are openly available in GenBank of NCBI at https://www.ncbi.nlm.nih.gov/ under the accession no. MW548267. The associated BioProject, SRA, and Bio-Sample numbers are PRJNA698244, SRR13589096, and SAMN17709251 respectively.
